# Development and application of a novel beta-tubulin genotyping tool reveals host-specific transmission cluster in *Balantioides coli*

**DOI:** 10.1371/journal.pntd.0013426

**Published:** 2025-08-14

**Authors:** Suhui Hu, Wen Zhang, Zhenzhen Liu, Junzhen Cheng, Qihao Zhang, Weifeng Qian, Min Zhang, Tianqi Wang, Wenchao Yan

**Affiliations:** 1 College of Animal Science and Technology, Henan University of Science and Technology, Luoyang, China; 2 Animal Disease Prevention and Control Center, Xuchang, China; Ege Universitesi Tip Fakultesi, TÜRKIYE

## Abstract

*Balantioides coli* is a zoonotic ciliated protozoan that infects humans and other mammals. Conventional and ITS-based genotyping approaches have limitations that hinder precise molecular epidemiological investigations. The objective of this study was to develop a new β-tubulin gene-based approach to enhance the detection and genotyping of *B. coli*. We performed single-cell isolation and whole-genome sequencing on two *B. coli* isolates from pigs and two from guinea pigs. We then used the β-tubulin gene sequences to design PCR primers for the new genotyping assay. We validated the assay using 56 ITS-confirmed *B. coli*-positive fecal DNA samples from pigs, cattle, sheep, and guinea pigs. Phylogenetic analyses were conducted using both β-tubulin and ITS sequences. The β-tubulin-based nested PCR assay exhibited 100% detection efficiency and greater specificity than ITS-based methods. Phylogenetic analysis of the β-tubulin gene sequences classified *B. coli* into three genotypes (I-III). Genotype III appears to be specific to guinea pigs. Genotypes I and II were found across multiple hosts, indicating potential cross-species transmission. Of the five full-length *B. coli* β-tubulin sequences obtained in this study, 264 polymorphic sites (19.8%) were identified, including both synonymous and non-synonymous mutations. Frequent recombination events within the β-tubulin locus were detected, indicating substantial genetic diversity. Therefore, the β-tubulin gene is a robust marker for genotyping and epidemiological studies of *B. coli*. The novel nested PCR assay overcomes the limitations of ITS-based methods and has produced data revealing previously unrecognized genetic diversity and host specificity patterns of *B. coli*.

## Author summary

*Balantioides coli*, a zoonotic ciliate protozoan, poses significant challenges in molecular epidemiology due to limitations of conventional subtyping methods targeting the internal transcribed spacer (ITS) region. In this study, we developed a nested PCR assay focusing on the β-tubulin gene, validated across 56 fecal samples from pigs, cattle, sheep, and guinea pigs. The assay demonstrated 100% detection efficiency and superior specificity compared to ITS-based approaches. Phylogenetic analysis delineated three β-tubulin genotypes: genotype III exhibited strict guinea pig host specificity, while genotypes I and II displayed cross-species transmission potential among pigs, cattle, and sheep. High genetic diversity and frequent recombination events underscored evolutionary adaptability. Non-synonymous mutations in genotype III correlated with reduced trophozoite motility, suggesting host-specific adaptation. This β-tubulin-based method overcomes ITS multicopy heterogeneity, enabling precise resolution of transmission clusters and host specificity patterns. Our findings advance surveillance strategies for *B. coli* in veterinary and public health frameworks, highlighting the utility of functional gene markers in combating neglected zoonotic pathogens.

## Introduction

*Balantioides coli* (previously *Balantidium coli*), a zoonotic ciliated protozoan parasite, represents the only known ciliate species capable of infecting humans, and various mammalian hosts including pig, cattle, buffalo, sheep, goats, rodents, and non-human primates [1,2]. *B. coli* is transmitted via the fecal-oral route, predominantly colonizing the host’s cecum and colon [2,3]. Infection occurs in humans and animals through direct or indirect ingestion of cyst-contaminated food or water. While immunocompetent hosts typically remain asymptomatic, immunocompromised individuals (e.g., Child or AIDS patients) may develop gastrointestinal manifestations, including diarrhea and malnutrition [1,4].

Currently, there is no standardized diagnostic method for *B. coli* detection, with conventional coproscopic examinations (e.g., flotation or sedimentation) remaining the primary diagnostic approach due to the parasite’s distinctive size and morphology [3]. However, microscopic methods suffer from limitations including low sensitivity, inability to assess genetic characteristics, and challenges in differentiating morphologically similar pathogens like *Buxtonella* spp. [1,4]. Molecular diagnostic tools are essential for elucidating genetic distinctions between *B. coli* and related ciliates, particularly given its taxonomic revisions. Originally classified as *Balantidium coli*, the organism was subsequently reassigned to genus *Neobalantidium* [5], and through ongoing taxonomic revisions has now been conclusively established under the genus *Balantioides* [6].

Recent advancements in molecular techniques have allowed for the investigation of different molecular characteristics of *B. coli.* Current method predominantly relies on the ITS region (ITS1-5.8S-rRNA-ITS2), which has revealed at least two genetic variants (A and B) with zoonotic potential attributed to variant A [7,8]. However, its location in a non-coding region and low evolutionary selective pressure result in a high mutation rate, compromising its accuracy. In this study, we identified the functional gene β-tubulin as a genotyping locus for *B. coli*. Due to its dual characteristics of sequence conservation and polymorphism, β-tubulin has been widely utilized in molecular typing of pathogenic microorganisms. For instance, β-tubulin sequence analysis has been applied to validate morphological classifications and clarify the phylogenetic position of *Fusarium tricinctum* [9,10]. In *Aspergillus fumigatus* subtyping, the integration of β-tubulin with cell surface protein (CSP) profiling has significantly enhanced the accuracy of genotype discrimination [11,12]. Recent studies have further demonstrated its utility in developing high-resolution multilocus sequence typing (MLST) tools for *Enterocytozoon bieneusi*, underscoring its cross-species applicability in subtyping [13].

In this study, we developed a novel nested PCR assay targeting the β-tubulin gene for molecular characterization of *Balantioides coli*. Compared to conventional ITS-based subtyping, this method demonstrates higher stability and specificity. The assay successfully amplified clinical isolates from diverse hosts including pigs, cattle, sheep, guinea pigs, suggesting broader applicability for epidemiological investigations.

## Materials and methods

### Ethics statement

The study’s protocol was approved by the Ethics Review Committee of Henna University of Science and Technology (Haust-025-M050632). All participants signed informed consent forms.

### Collection of *Balantioides coli*-positive samples

DNA preparations from 56 fecal samples were used in this study, including those from pigs (n = 32), cattle (n = 1), sheep (n = 5), and guinea pig (n = 10) (S1 Table). These samples were previously collected on 19 farms in 10 regions of China and identified as *B. coli*-positive by PCR and sequence analysis of the ITS gene. Genomic DNA from all 56 samples was extracted using Stool DNA Kit (Omega Biotek, USA) following the manufacturer’s instructions and then stored at -20 °C.

### Isolation of *Balantioides coli* trophozoites

The porcine *Balantioides coli* isolates (B1184, B1186) were obtained from diarrheic fecal samples of a pig farm in Luoyang China, with trophozoites confirmed by microscopy before cultivation [14]. Guinea pigs were purchased from pet markets and immunosuppressed with dexamethasone via drinking water for one week. The cecal contents from diarrheic guinea pigs (showing abundant trophozoites upon microscopic examination) were collected to isolate strains T404 and T406 for subsequent culture [14]. Sequence analysis of the ITS locus identified genetic variant A in both isolates derived from pigs, whereas the two guinea pigs derived isolates exhibited significant divergence from both variants A and B.

### In vitro culture of trophozoites

The isolated trophozoites were cultured in DMEM medium (detailed protocol see reference [14]). Approximately two-thirds of the supernatant was removed and replaced with an equal volume of fresh medium daily. Subculture was performed during peak growth phases. After 3–4 passages when contaminants were significantly reduced, trophozoites were purified using single-cell micromanipulation. To characterize the in vitro growth dynamics of porcine- and guinea pig-derived *Balantioides coli*, the 3 mL culture suspension was gently homogenized before a 30 μL suspension was transferred onto a glass slide for microscopic enumeration at 10 × magnification, with total trophozoite counts in the complete culture system calculated by proportional extrapolation.

### Single-cell micromanipulation of trophozoites

The single-cell isolation procedure was performed as follows: Glass capillaries were heated at the center in an alcohol lamp flame for approximately 3 s until softened, then rapidly drawn to prepare fine microneedles. The blunt end of the capillary was inserted to half-depth into a mouth pipette assembly. For isolation, 30 μL of trophozoite suspension was placed in a culture dish alongside three droplets of PBS washing buffer. Under a stereomicroscope, target trophozoites were aspirated by gentle mouth suction and sequentially transferred through the PBS droplets for a minimum of three washes to remove contaminants. The purified trophozoites were collected in EP tubes, with 2,000–3,000 trophozoites isolated per strain for subsequent sequencing.

### Whole-genome sequencing and beta-tubulin gene screening

Using the previously sequenced genome of porcine *B. coli* strain P011 (GenBank accession: GWHBOZN00000000) as reference, this isolate was identified as genetic variant B based on ITS locus analysis. In this study, all purified trophozoite samples were subjected to Illumina NovaSeq high-throughput sequencing and assembly at Shanghai Majorbio Bio-pharm Technology Co., Ltd. The gene sequence was visualized and annotated using IGV software [15].

Following the acquisition of draft genomic sequences, we initially employed the Fasta Extract function in TBtools to retrieve and extract sequences exhibiting high similarity to the reference gene EVM0007701.1 (β-tubulin gene) from four isolated strains. We subsequently performed comparative analysis of the amino acid sequences encoded by the β-tubulin genes from five isolated strains, predicted conserved domains using the Conserved Domain Database (CDD; https://www.ncbi.nlm.nih.gov/structure/cdd), and compared these domains with those from β-tubulin genes of 13 ciliates.

### Genotyping primer design and nest PCR amplification

The gene sequence was visualized and annotated using IGV software [15]. Complete β-tubulin gene sequences were extracted using the Fasta Extract tool in TBtools [16]. Nest-PCR primers were designed using Primer Premier 5 software. The β-tubulin gene sequences ware amplified with the external PCR primer pair (F1: 5’-AACTGGGCTAAGGGACACTA-3’ and R1: 5’-CTCCATTTCGTCCATACCTT-3’) and the internal primer pair (F2: 5’-GACCTTCGCTGTCTTCCC-3’ and R2: 5’-TTCTCCGGTGTACCAATGT-3’).

Nested PCR reaction conditions were conducted as below. The first PCR reaction was conducted with the following steps: 5 min at 94 °C followed by 35 cycles of 94 °C for 40 s, 53 °C for 40 s, and 72 °C for 1 min, followed by a final extension for 10 min at 72 °C. The second reaction used a modified program: comprising 94 °C for 5 min followed by 35 cycles of 94 °C for 40 s, 53 °C for 40 s, and 72 °C for 55 s, followed by a final extension at 72 °C for 10 min.

The nested PCR amplification was performed in a two-step reaction system as follows: (1) Primary PCR: Each 25 μL reaction mixture contained 12.5 μL of 2 × Taq-PCR-StarMix, 1 μL each of forward and reverse outer primers (F1/R1), 9.5 μL of ddH₂O, and 1 μL of genomic DNA template. (2) Secondary PCR: The secondary reaction (25 μL total volume) utilized 12.5 μL of 2 × Taq-PCR-StarMix, 1 μL each of nested primers (F2/R2), 9.5 μL of ddH₂O, and 1 μL of diluted primary PCR product as the template. Both rounds of amplification were conducted under identical thermal cycling conditions, with primer-specific annealing temperatures optimized for each primer pair.

### Sequencing and phylogenetic analysis

The positive PCR products were directly sequenced bidirectionally with Sanger sequencing by a commercial sequencing company (GENEWIZ, Suzhou, China). The nucleotide sequences of *B. coli* were aligned using Clustal X 2.1 [17] with default parameters. Phylogenetic analysis was conducted in MEGA X [18] by constructing maximum likelihood (ML) trees based on the General Time Reversible (GTR) model. Node support was assessed through 1000 bootstrap replicates, with values more than 70% considered statistically significant for clade support Final visualization and annotation of the phylogenetic tree were achieved using the Interactive Tree of Life (iTOL) platform (https://itol.embl.de/). Potential recombination events (Rm) were detected and analyzed using DNAsp v 6.12 (http://www.ub.edu/dnasp/).

### Nucleotide sequence accession numbers

The β-tubulin gene sequences of *B. coli* obtained in this study (B1184, B1186, T404, T406, P011) from guinea pig and pig hosts were deposited in GenBank under accession numbers PV609776-PV609780.

## Results

### In vitro growth characteristics of *B. coli* trophozoites

**Fig 1:**
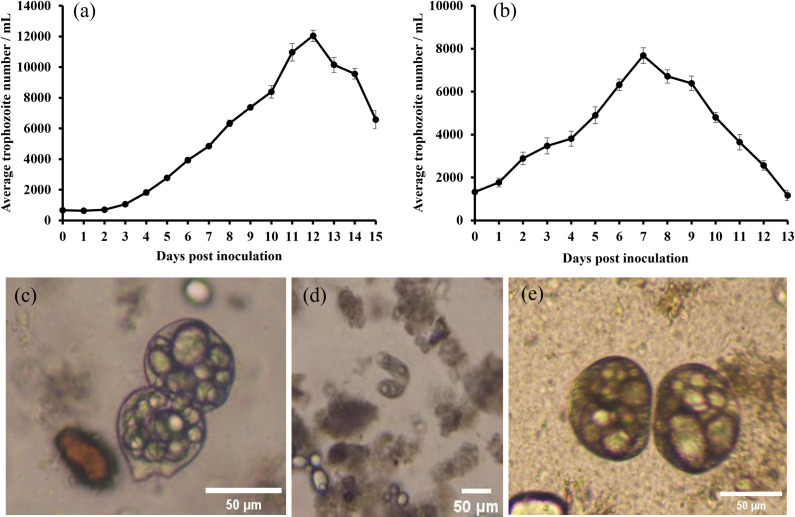
The growth of pig (a) and guinea pig (b) derived *B. coli* trophozoites in DMEM medium. Observation of binary fission (c) and conjugation (d) in pig-derived *B. coli* trophozoites, but only binary fission in guinea pig-derived isolates during growth (e).

The porcine-derived *B. coli* trophozoites grew well in the modified DMEM medium, reaching a peak density of 12,040 trophozoites/mL on day 12 (Fig 1a). During cultivation, both conjugation and binary fission were observed as the reproductive modes (Fig 1c and 1d).

In contrast, the guinea pig-derived *B. coli* trophozoites reached their peak density (7,678 trophozoites/mL) on day 7 (Fig 1b). Compared to the porcine-derived trophozoites, they exhibited slower motility and a more rounded shape in the modified DMEM medium. Only binary fission was observed as the reproductive mode (Fig 1e).

### Conserved structure of *B. coli* β-tubulin gene

**Fig 2:**
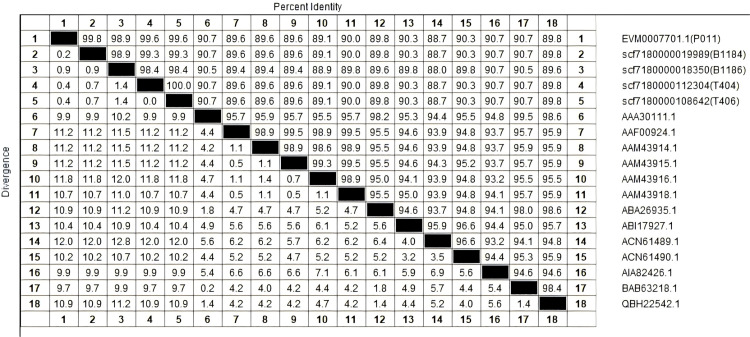
Homology analysis of complete amino acid sequence of β-tubulin.

**Fig 3:**
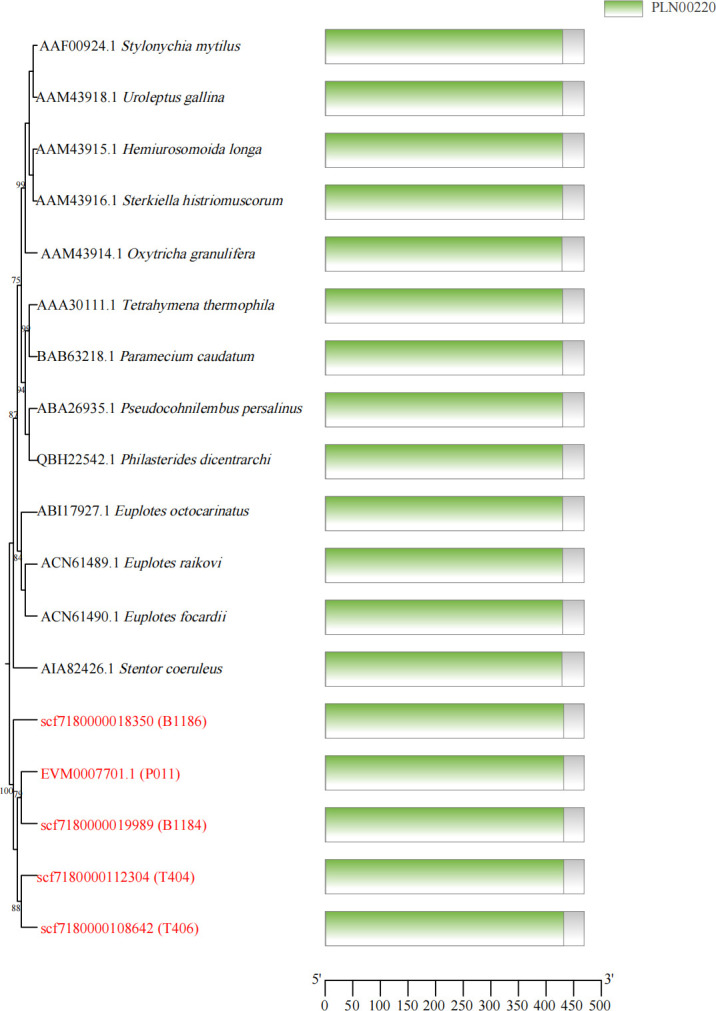
β-tubulin protein domains of different species origin.

Although the sequenced genomes exhibited limited alignment coverage to the reference genome, β-tubulin gene fragments were successfully identified across four sequenced datasets. To validate these five candidate β-tubulin genes, comparative analyses including homology assessment (Fig 2) and domain alignment (Fig 3) were performed against β-tubulin genes from 13 ciliated pathogens. These integrated analyses confirmed their classification as authentic β-tubulin genes, revealing conserved domain architectures characteristic of this evolutionarily preserved protein family.

### Sequence polymorphisms in β-tubulin gene

The complete β-tubulin gene (EVM0007701.1) was identified in scf7180000019989 of the reference genome sequences of *B. coli* (GWHBOZN00000000). The resequencing data from all four isolates showed alignment rates below 10.0% when mapped to the reference genome. BLAST analysis revealed the absence of 18S and ITS genes in the guinea pig-derived isolate, suggesting potential technical issues in its sequencing results. Notably, β-tubulin gene sequences were successfully identified in all four isolates. Specifically, the β-tubulin genes of two pig-derived isolates measured 1136 bp and 1138 bp, respectively, while the guinea pig-derived isolate exhibited a β-tubulin gene length of 1143 bp (Table 1). In summary,

**Table 1:** Location information of β-tubulin sequences in the draft whole genomes.

Sample ID	Sequence fragment	Start-end nucleotide position	Sequence length (bp)
B1184	scf7180000019989	112 bp - 1449 bp	1338
B1186	scf7180000018350	124 bp - 1459 bp	1336
T404	scf7180000112304	818 bp - 2160 bp	1343
T406	scf71800000108642	571 bp - 1913 bp	1343

comparative analysis of β-tubulin sequences from five genomes identified 264 polymorphic sites (positions 1–1336 bp), with the polymorphic site frequency of 19.8%

(S1 Fig). Phylogenetic analysis based on β-tubulin gene sequences revealed that all sequences could be classified into three distinct clades: the pig-derived isolates B1184 and B1186 formed one cluster, the reference genome sequence (EVM0007701.1) constituted a separate branch, while the guinea pig-derived isolates T404 and T406 formed another cluster. Based on their genetic clustering characteristics, these clades were designated as genotype I, genotype II, and genotype III, respectively (Fig 4).

**Fig 4:**
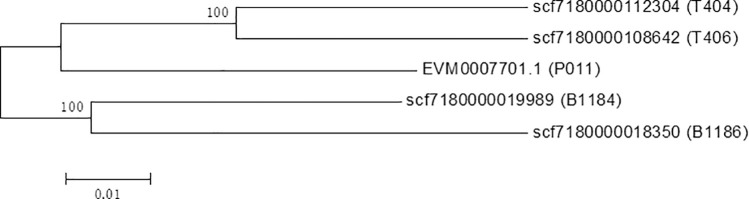
Phylogenetic analysis based on full-length β-tubulin sequences.

Using the Expasy-Translate online tool (https://web.expasy.org/translate/), the β-tubulin gene sequences from four isolates were translated into amino acid sequences with ATG as the start codon. Comparative analysis of these sequences against the reference genome revealed that the majority of polymorphic sites corresponded to synonymous mutations, while only five positions exhibited non-synonymous substitutions (S2 Fig). Specifically, amino acid differences were identified at positions 155 (isoleucine, I vs. valine, V) and 169 (I vs. V), 248 (serine, S vs. alanine, A), 438 (glutamic acid, E vs. glycine, G), and 442 (G vs. A).

### Amplification efficiency of β-tubulin primers

**Fig 5:**
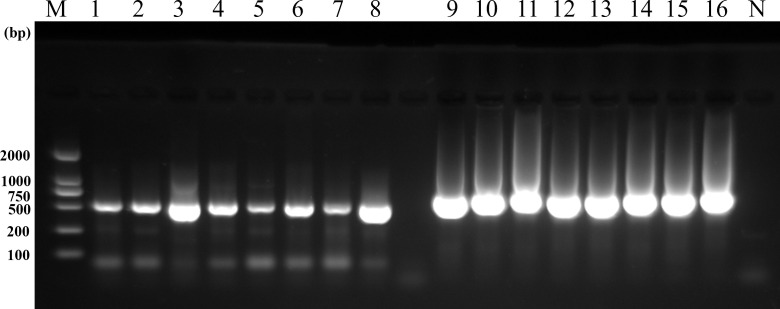
Comparison of PCR amplification results for β-tubulin and ITS loci (M: Marker; N: Negative control). Lanes 1-8 show the ITS locus, and lanes 9-16 show the β-tubulin locus. Eight samples were selected for analysis, in the following order from left to right: pig (n = 3), sheep (n = 2), cattle (n = 1), and guinea pig (n = 2).

Nested PCR targeting the β-tubulin gene was performed on 56 *B. coli* isolates from different hosts, achieving a 100% positivity rate. Comparative electrophoresis analysis revealed that the β-tubulin gene exhibited higher efficiency than the ITS region. Notably, the β-tubulin amplicons showed significantly stronger band intensity than ITS amplicons in the same samples (Fig 5).

### Phylogenetic relationship of *Balantioides coli* isolates based on β-tubulin sequences

**Fig 6:**
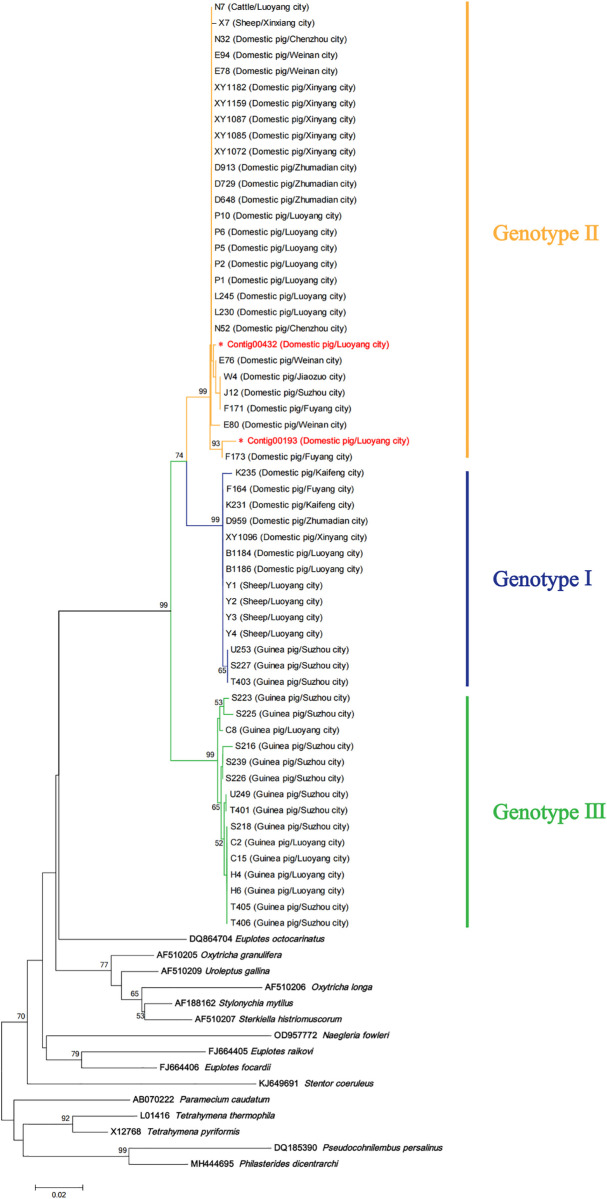
Phylogenetic tree constructed based on β-tubulin sequences.

The β-tubulin gene sequences of 56 isolates were aligned and subjected to phylogenetic analysis. The resulting evolutionary tree revealed that all samples formed three distinct clades (genotype I-III) at this locus, with significant genetic divergence from the β-tubulin genes of other ciliate species (Fig 6). Further analysis demonstrated

that genotype I comprised a mixed-host cluster including swine-derived (B1184, B1186, etc.), sheep-derived, and guinea pig-derived isolates, suggesting potential cross-species transmission capacity. Genotype II, which included the reference genome, clustered with certain porcine-, bovine-, and sheep-derived strains. Notably, genotype III exclusively contained guinea pig-derived isolates, demonstrating host specificity. Sequence alignment revealed 50 haplotypes among the 56 sequences, with haplotype diversity reaching 0.98. In a DnaSP analysis, 8 potential recombination events were identified among all the three genotypes. During sequence analysis, BLAST alignment revealed that strain P011 harbored two distinct ITS sequences corresponding to Contig00191 and Contig00364, respectively. This result indicates the presence of polymorphisms at the ITS locus within the same isolate and also suggests the possibility of mixed infection in the sample.

### Phylogenetic relationship of *Balantioides coli* isolates based on ITS sequences

**Fig 7:**
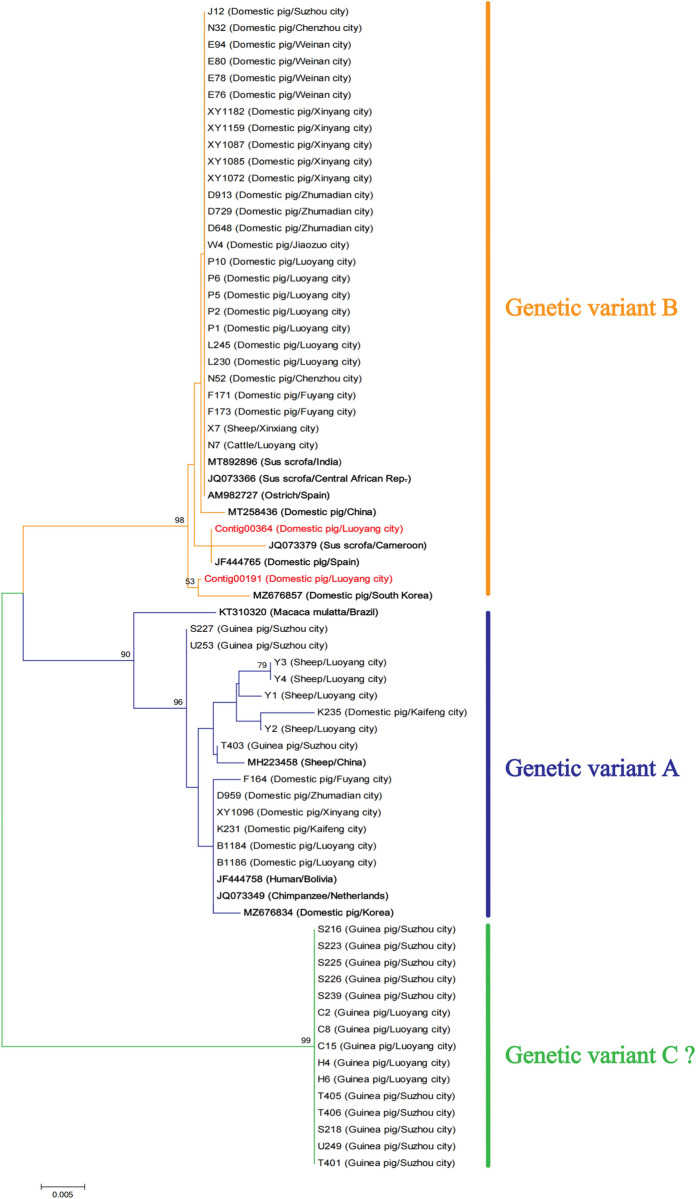
Phylogenetic tree constructed based on ITS sequences.

Phylogenetic analysis based on the ITS sequences of 56 samples showed that all samples could be divided into three clades (Fig 7). Among these, 27 samples (25 from pigs, 1 from cattle, and 1 from sheep) clustered with the reference genetic variant B sequences (reference sequences JQ073366, AM982727, and others). Fourteen samples (7 from pigs, 3 from guinea pigs, and 4 from sheep) grouped with genetic variant A (reference sequences MZ676834, JQ073349, and others). Additionally, 15 guinea pig-derived samples formed an independent clade with high bootstrap support (99.0%), which showed significant genetic divergence from the known variants and was tentatively designated as the novel genetic variant C.

### Comparative phylogenetic analysis of *Balantioides coli* based on ITS and β-tubulin loci

Phylogenetic analysis of 56 isolates based on ITS and β-tubulin gene loci revealed both agreement and discrepancy between the two molecular markers. The classification results demonstrated remarkable consistency, both markers segregated the isolates into three corresponding groups, with ITS variants A, B, and C matching β-tubulin genotypes I, II, and III, respectively. Notably, the subgroup distributions were identical between both systems, containing 14 isolates in variant A/genotype I, 27 in variant B/genotype II, and 15 in variant C/genotype III. This complete correspondence validates the reliability of both markers for classification within our study system.

However, distinct patterns of sequence polymorphism were observed between the loci: variant A exhibited sequence polymorphism, while genotype I showed sequence conservation. Conversely, in variant C/genotype III, guinea pig-derived isolates displayed greater sequence polymorphism at the β-tubulin locus. These differential polymorphism patterns suggest that although the two markers produce congruent classification results, they provide complementary information regarding genetic diversity. The combined use of both markers offers more comprehensive subtyping information and enhances the accuracy of phylogenetic inferences.

## Discussion

A novel β-tubulin gene-based genotyping method was developed to classify *B. coli* into three genotypes (I-III). Using single-cell isolation, we obtained two pig-derived and two guinea pig-derived isolates, and successfully acquired β-tubulin gene sequences through next-generation sequencing. Based on molecular marker, we established a PCR assay capable of detecting *B. coli* from pigs, cattle, sheep, and guinea pigs. Phylogenetic analysis revealed that genotypes I and II exhibited host adaptability, whereas genotype III displayed strict host specificity. This genotyping approach provides a valuable tool for molecular epidemiological studies of *B. coli*.

This study establishes the functional β-tubulin gene as a novel molecular marker for *B. coli* genotyping. It may overcome limitations of ITS-based methods, which struggle to resolve genetic diversity due to multicopy variation and high mutation rates. The ITS region, a noncoding segment of ribosomal DNA between 18S, 5.8S, and 28S rRNA genes [19], is widely used for parasite differentiation [20–22]. However, ITS multicopy heterogeneity can distort results. For example, *Cryptosporidium parvum* and *B. coli* show two ITS genotypes within a single genome, indicating intragenomic polymorphisms rather than true speciation [23,24]. This finding is consistent with our data. For instance, isolate P011 harbored two distinct ITS sequences (Contig00191 and Contig00364), indicating that ITS analysis alone cannot reliably differentiate mixed infections from naturally occurring polymorphisms. In contrast, β-tubulin combines conserved functional domains with localized polymorphism [25]. Its conserved regions enable universal primer design (100% detection rate for 56 samples), while 19.8% polymorphic sites (S1 Fig) provide high-resolution genetic signals. Critically, β-tubulin mutations (e.g., Ala → Ser at position 248) may be associated with phenotypes such as reduced trophozoite motility, as we have observed lower motility in guinea pig-derived isolates compared to those from pigs, although further validation is required This functional-evolutionary dual analysis resolves ITS limitations, advancing *B. coli* genotyping and transmission research.

Phylogenetic analysis based on the β-tubulin gene classified *B. coli* into three genotypes (I-III), revealing significant associations between host adaptation and genetic divergence. The cross-host transmission capabilities of genotypes I and II may be linked to their high-frequency synonymous mutations (19.8% polymorphic sites), which likely maintain microtubule structural stability to enhance host adaptability. This phenomenon aligns with observations in *Pseudomonas fluorescens*, where synonymous mutations drive adaptive evolution [26]. In contrast, genotype III harbors critical non-synonymous mutations, such as the Ala to Ser substitution at position 248, which may disrupt the negative charge distribution of β-tubulin, thereby impairing motility and restricting host range. The broad host adaptability of genotypes I and II suggests significant zoonotic potential, highlighting the need for a β-tubulin-based surveillance system in livestock-dense regions. Conversely, the host specificity of genotype III likely stems from adaptive evolution to the unique intestinal microenvironment of rodents. The higher dehydration level of rodent cecal and colonic contents [27] results in distinct gut microbiota composition and metabolic profiles. Given *B. coli*’s obligate dependence on symbiotic microbiota for essential nutrients, these microenvironmental disparities may impose selective pressures that shape genomic divergence, as evidenced by genotype III-specific mutations [28,29].

The nested PCR assay targeting the β-tubulin gene developed in this study provides an effective tool for investigating the transmission dynamics of *B. coli*. Our method demonstrated 100% amplification efficiency when applied to 56 ITS-positive DNA samples derived from various host species (Fig 4), attributable to the enhanced sensitivity and specificity conferred by the nested PCR design. However, due to sample limitations (primarily obtained from swine, cattle, sheep, and guinea pigs), the current dataset cannot fully validate the universal applicability of this assay across all potential host species. Future studies should incorporate expanded sample collections (particularly from susceptible primate hosts) and conduct systematic epidemiological surveillance to further evaluate the robustness of this detection system.

The high genetic diversity of *B. coli* may be attributed to the frequent occurrence of genetic recombination. In this study, 121 potential recombination events were detected within the β-tubulin gene sequences, further supporting the notion of high-frequency genetic variation at this locus. Previous reports have indicated that the high prevalence [1,3,4,30] and prolonged duration of *B. coli* infections may greatly facilitate inter-isolate genetic recombination, potentially leading to the emergence of novel subtypes. This phenomenon has been well-documented in apicomplexan parasites (e.g., *Plasmodium*, *Toxoplasma*, and *Cryptosporidium*), where genetic recombination influences their adaptive evolution and zoonotic potential. Specifically, in high-transmission regions, *Plasmodium falciparum* frequently undergoes recombination due to multi-lineage co-infections, accelerating the spread of drug-resistant genes. Similarly, hybridization between different *Toxoplasma gondii* strains can generate offspring with enhanced virulence, while recombination among *Cryptosporidium* subtypes may produce novel variants with altered host adaptability. Collectively, these findings highlight the pivotal role of genetic recombination in parasitic evolution, including the development of drug resistance, virulence enhancement, and host adaptation [31–35].

## Conclusions

This study successfully developed a novel β-tubulin based nested PCR method for *B. coli* genotyping. The assay demonstrated 100% detection efficiency and higher specificity when compared to ITS-based method. Three distinct genotypes were identified through β-tubulin analysis, each showing characteristic host adaptation patterns. Genotype III showed strict specificity to guinea pigs, whereas genotypes I and II exhibited potential for cross-species transmission among pigs, cattle and sheep. The β-tubulin marker proved to be a stable genetic marker that effectively addressed the limitations associated with ITS markers, including multicopy heterogeneity and high mutation rates. This high-resolution genotyping system provides an improved tool for molecular epidemiological surveillance, enabling more accurate assessment of zoonotic risks. Standardized application of this assay across different laboratories will facilitate comparative studies and enhance management of *B. coli*-related veterinary and public health concerns.

## Supporting information

S1 Fig Alignment of β-tubulin gene sequences. (TIF)

S2 Fig Alignment of β-tubulin amino acid sequences. (TIF)

S1 Table Information of DNA from *B. coli* positive fecal samples. (DOCX)
