# Supplementary figures and images for: Development and application of a novel beta-tubulin genotyping tool reveals host-specific transmission cluster in Balantioides coli
Source: PLoS Negl Trop Dis. 2025 Aug 14;19(8):e0013426. doi: 10.1371/journal.pntd.0013426 (PMC12352754; doi:10.1371/journal.pntd.0013426)

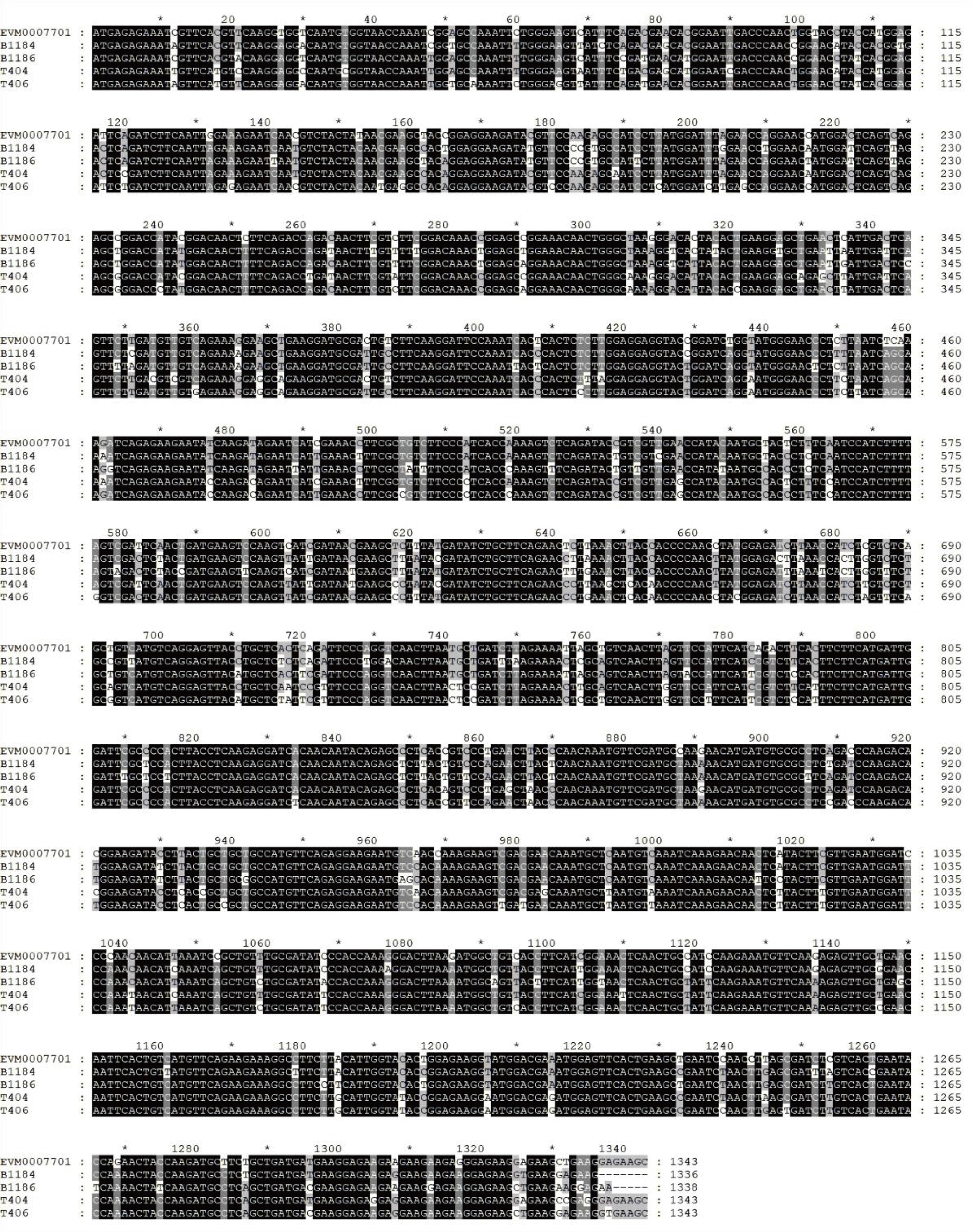

Supplement: S1 Fig — (TIF) [file pntd.0013426.s001.tif]

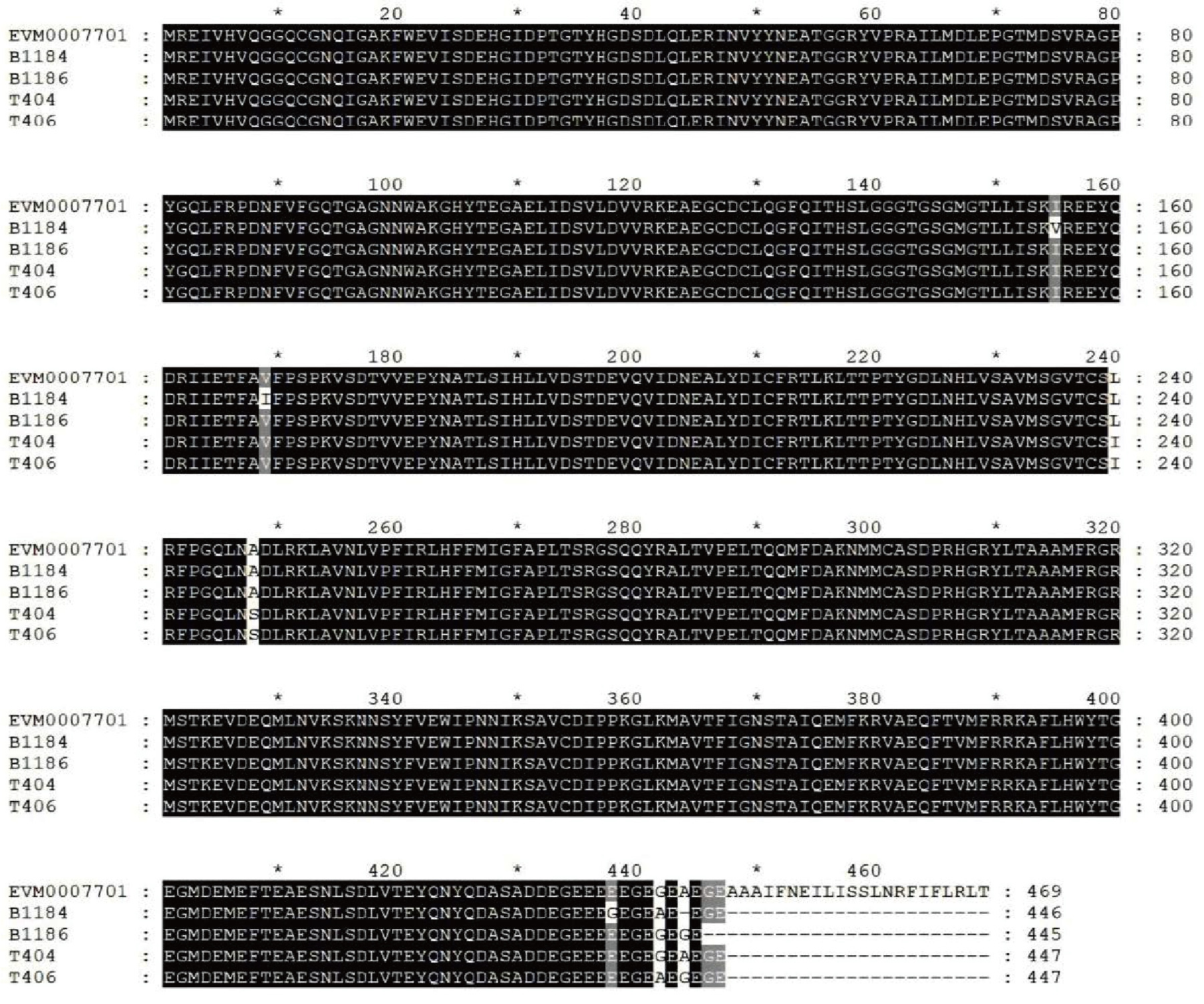

Supplement: S2 Fig — (TIF) [file pntd.0013426.s002.tif]
