# Supplementary material for: Development and application of a novel beta-tubulin genotyping tool reveals host-specific transmission cluster in Balantioides coli
Source: PLoS Negl Trop Dis. 2025 Aug 14;19(8):e0013426. doi: 10.1371/journal.pntd.0013426 (PMC12352754; doi:10.1371/journal.pntd.0013426)
Supplement: S1 Table — (DOCX) [file pntd.0013426.s003.docx]

Supplementary Tab. 1 Information of DNA from *Balantioides coli* positive fecal samples

| No. | Sample ID | Host | Location | No. | Sample ID | | Host | Location |
| --- | --- | --- | --- | --- | --- | --- | --- | --- |
| 1 | L230 | Pig | Luoyang | 29 | K231 | Pig | | Kaifeng |
| 2 | L245 | Pig | Luoyang | 30 | K235 | Pig | | Kaifeng |
| 3 | P1 | Pig | Luoyang | 31 | B1184 | Pig | | Luoyang |
| 4 | P2 | Pig | Luoyang | 32 | B1186 | Pig | | Luoyang |
| 5 | P5 | Pig | Luoyang | 33 | N7 | Cattle | | Luoyang |
| 6 | P6 | Pig | Luoyang | 34 | X7 | sheep | | Xinxiang |
| 7 | P10 | Pig | Luoyang | 35 | Y1 | sheep | | Luoyang |
| 8 | W4 | Pig | Jiaozuo | 36 | Y2 | sheep | | Luoyang |
| 9 | D648 | Pig | Zhumadian | 37 | Y3 | sheep | | Luoyang |
| 10 | D729 | Pig | Zhumadian | 38 | Y4 | sheep | | Luoyang |
| 11 | D913 | Pig | Zhumadian | 39 | S216 | Guinea pig | | Suzhou |
| 12 | D959 | Pig | Zhumadian | 40 | S218 | Guinea pig | | Suzhou |
| 13 | XY1072 | Pig | Xinyang | 41 | S223 | Guinea pig | | Suzhou |
| 14 | XY1085 | Pig | Xinyang | 42 | S225 | Guinea pig | | Suzhou |
| 15 | XY1087 | Pig | Xinyang | 43 | S226 | Guinea pig | | Suzhou |
| 16 | XY1096 | Pig | Xinyang | 44 | S227 | Guinea pig | | Suzhou |
| 17 | XY1159 | Pig | Xinyang | 45 | S239 | Guinea pig | | Suzhou |
| 18 | XY1182 | Pig | Xinyang | 46 | U249 | Guinea pig | | Suzhou |
| 19 | E76 | Pig | Weinan | 47 | U253 | Guinea pig | | Suzhou |
| 20 | E78 | Pig | Weinan | 48 | C2 | Guinea pig | | Luoyang |
| 21 | E80 | Pig | Weinan | 49 | C8 | Guinea pig | | Luoyang |
| 22 | E94 | Pig | Weinan | 50 | C15 | Guinea pig | | Luoyang |
| 23 | J12 | Pig | Suzhou | 51 | H4 | Guinea pig | | Luoyang |
| 24 | N32 | Pig | Chenzhou | 52 | H6 | Guinea pig | | Luoyang |
| 25 | N52 | Pig | Chenzhou | 53 | T401 | Guinea pig | | Suzhou |
| 26 | F164 | Pig | Fuyang | 54 | T403 | Guinea pig | | Suzhou |
| 27 | F171 | Pig | Fuyang | 55 | T405 | Guinea pig | | Suzhou |
| 28 | F173 | Pig | Fuyang | 56 | T406 | Guinea pig | | Suzhou |

Note: The initial letter of the sample ID indicates the different farm locations
